# Supplementary material for: Cold exposure induces dynamic changes in circulating triacylglycerol species, which is dependent on intracellular lipolysis: A randomized cross-over trial
Source: eBioMedicine. 2022 Nov 11;86:104349. doi: 10.1016/j.ebiom.2022.104349 (PMC9663865; doi:10.1016/j.ebiom.2022.104349)
Supplement: Supplementary Figures S1–S3 [file mmc1.docx]

**Supplemental figures**

**Cold exposure induces dynamic changes in circulating triacylglycerol species, which is dependent on intracellular lipolysis**

Maaike E. Straat^1,2,#^, Lucas Jurado-Fasoli^3,#^, Zhixiong Ying^1,2^, Kimberly J. Nahon^1,2^, Laura G.M. Janssen^1,2^, Mariëtte R. Boon^1,2^, Gernot F. Grabner^5^, Sander Kooijman^1,2^, Robert Zimmermann^5^, Martin Giera^4^, Patrick C.N. Rensen^1,2*^, Borja Martinez-Tellez^1,2,‡^

1. Division of Endocrinology, Department of Medicine, Leiden University Medical Center, Leiden, the Netherlands.

2. Einthoven Laboratory for Experimental Vascular Medicine, Leiden University Medical Center, Leiden, the Netherlands.

3. PROmoting FITness and Health through physical activity research group (PROFITH), Sport and Health University Research Institute (iMUDS), University of Granada, Granada, Spain.

4. Center for Proteomics and Metabolomics, Leiden University Medical Center, Leiden, The Netherlands.

5. Institute of Molecular Biosciences, University of Graz, Graz, Austria.

^‡^Present address: Department of Education, Faculty of Education Sciences and SPORT Research Group (CTS-1024), CERNEP Research Center, University of Almería, Almería, Spain.

***Corresponding** **author:** Patrick C.N. Rensen, P.C.N.Rensen@lumc.nl, Albinusdreef 2, 2333 ZA, Leiden, The Netherlands

**^#^These authors share first authorship**


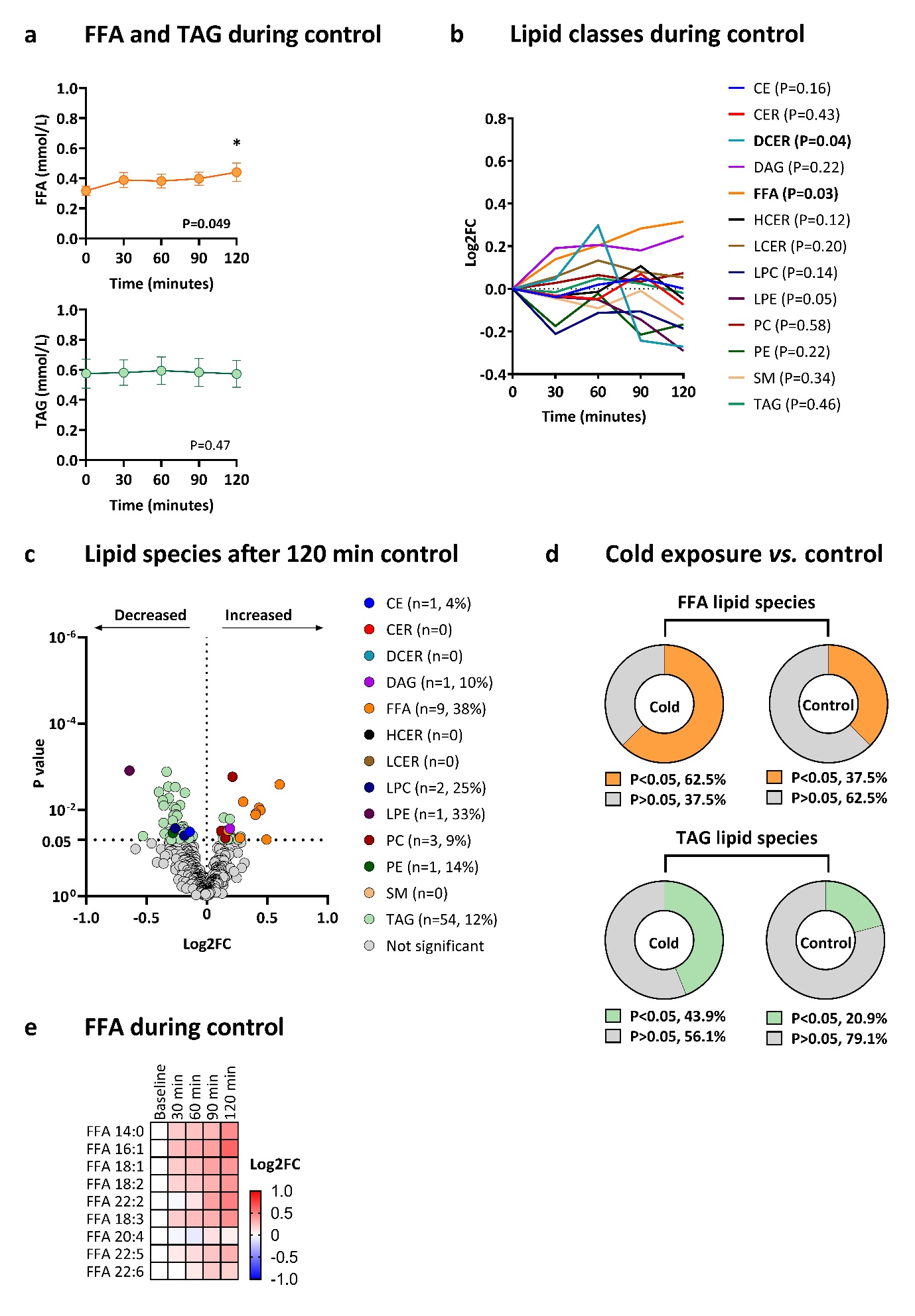


**Figure S1. Lipidomic changes during the control experiment without personalized cold exposure**

(**a**) Dynamic changes in total free fatty acid and triacylglycerol concentration during the control experiment, as measured with enzymatic assays. Data is presented as mean and standard error of the mean. P values are obtained from ANOVA repeated measures. *P<0.05; compared to baseline (*i.e.*, 0 min). (**b**) Dynamic changes in lipid classes during the control experiment. Data is presented as the log2 fold change (log2FC) relative to baseline (*i.e.*, 0 min). ANOVA repeated measures were used to compare the different timepoints. (**c**) Volcano plot showing the change of individual lipid species after 120 min during the control experiment exposed to room temperature. The X-axis shows the log2FC between 120 min *vs.* baseline, the Y-axis shows the P value. P values are obtained from paired Student’s t test. Values between brackets indicate the absolute number and the percentage of lipid species within the lipid class that were modulated. (**d**) FFA and TAG species that significantly dynamically changed during cold exposure (left) or during the control experiment (right). P values are obtained from ANOVA repeated measures, percentages indicate the lipid species within the lipid class that were modulated. (**e**) Heatmap of FFA species that significantly dynamically changed during the control experiment. The color of the squares represents the log2FC relative to baseline. FFAs are sorted by the number of double bonds. ANOVA repeated measures were used to compare the different timepoints. CE, cholesteryl esters; CER, ceramides; DAG, diacylglycerols; DCER, dihydroceramides; FFA, free fatty acids; FC, fold change; HCER, hexosylceramides; LCER, lactosylceramides; LPC, lysophosphatidylcholine; LPE, lysophosphatidylethanolamine; PC, phosphatidylcholine; PE, phosphatidylethanolamine; SM, sphingomyelin; TAG, triacylglycerols.

**
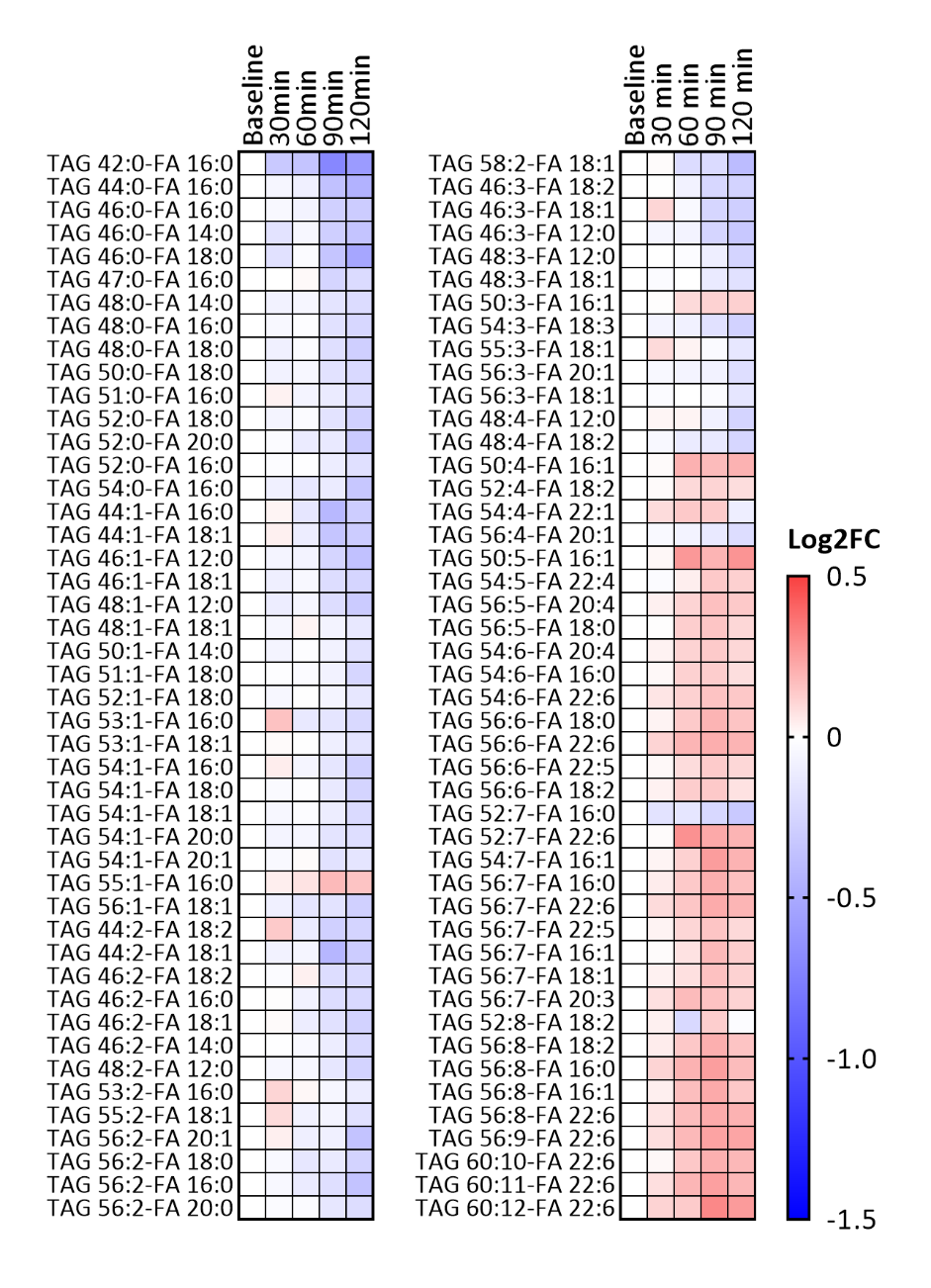
**

**Figure S2. Triacylglycerol (TAG) species during the control experiment without personalized cold exposure**

Heatmap of TAG species that significantly dynamically changed during the control experiment. The color of the squares represents the log2 fold change (log2FC) relative to baseline. TAGs are sorted by the number of double bonds. ANOVA repeated measures were used to compare the different timepoints. FA, Fatty acid; FC, fold change; TAG, triacylglycerol.


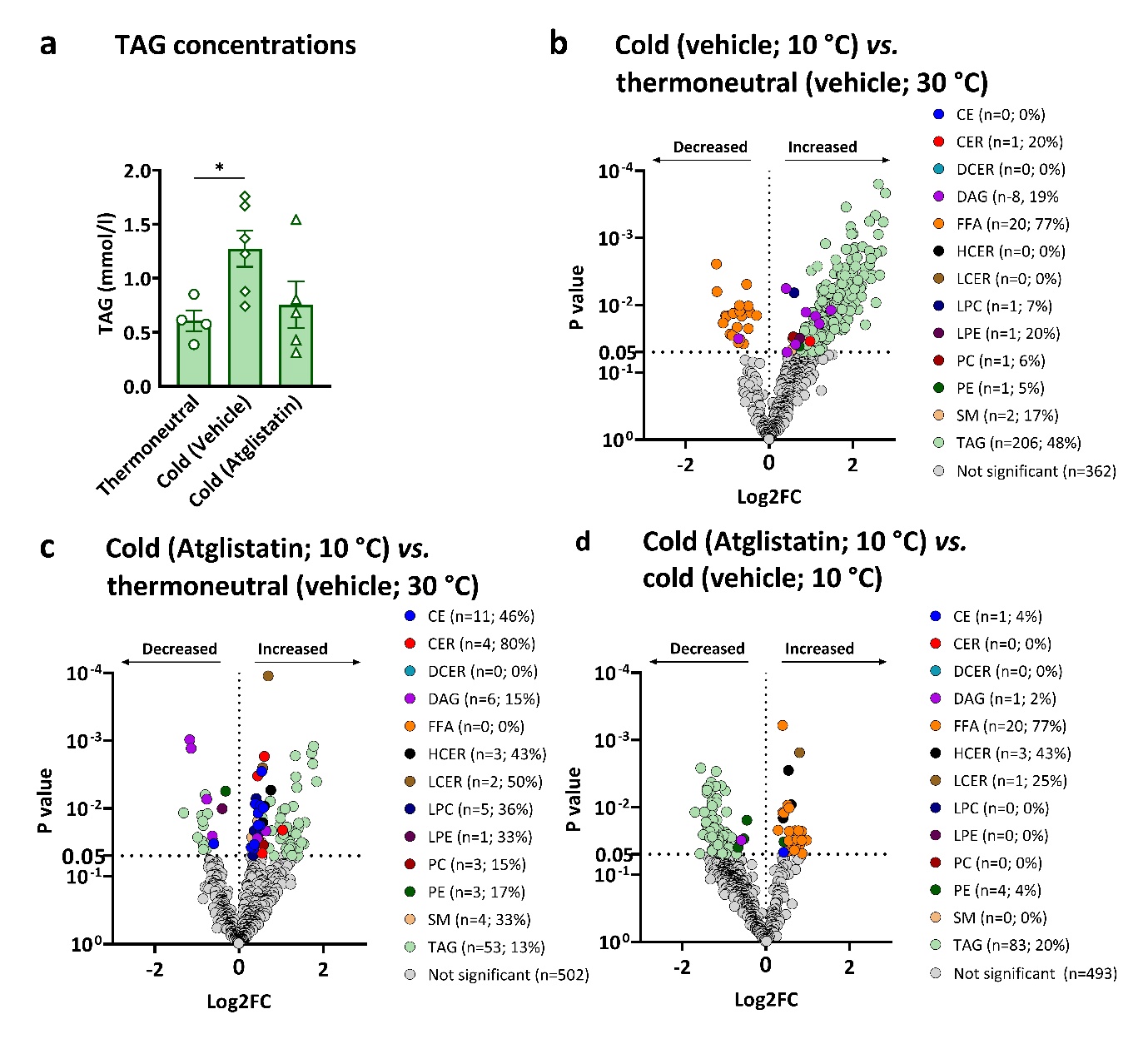


**Figure S3. The effect of cold exposure on individual lipid species in mice**

(**a**) Total triacylglycerol (TAG) concentration of mice just after the intervention, as measured with an enzymatic assay. Data is presented as mean and standard error of the mean. P value is obtained from Mann Whitney U test. (**b-d**) Volcano plot showing the differences in lipid species between mice exposed to cold *vs.* mice kept at thermoneutrality (**b**) (n=6 each group; both treated with vehicle); between mice exposed to cold (n=6; treated with Atglistatin) *vs.* mice kept at thermoneutrality (treated with vehicle) (**c**); and between mice exposed to cold (n=6; treated with Atglistatin) *vs.* mice exposed to cold (n=6; treated with vehicle) (**d**). The X-axis shows the log2 fold change (log2FC) between the groups, the Y-axis shows the P value. P values are obtained from unpaired Student’s t test. Values between brackets indicate the absolute number and the percentage of lipid species within the lipid class that were modulated by cold exposure (**b**, **c**), and by Atglistatin treatment (**d**). CE, cholesteryl esters; CER, ceramides; DAG, diacylglycerols; DCER, dihydroceramides; FFA, free fatty acids; FC, fold change; HCER, hexosylceramides; LCER, lactosylceramides; LPC, lysophosphatidylcholine; LPE, lysophosphatidylethanolamine; PC, phosphatidylcholine; PE, phosphatidylethanolamine; SM, sphingomyelin; TAG, triacylglycerols.
