# Supplementary material for: Cold exposure induces dynamic changes in circulating triacylglycerol species, which is dependent on intracellular lipolysis: A randomized cross-over trial
Source: eBioMedicine. 2022 Nov 11;86:104349. doi: 10.1016/j.ebiom.2022.104349 (PMC9663865; doi:10.1016/j.ebiom.2022.104349)
Supplement: Supplementary data [file mmc2.pdf]

The effect of Mirabegron on brown adipose tissue in healthy young white Caucasian and South Asian men

Versie 2: March 2016

Protocolnummer: ….

Verrichter: Dr. I.M. Jazet

Uitvoerder: Dr. I.M. Jazet

Contactpersoon: Dr. I.M. Jazet
Afdeling Algemene Interne Geneeskunde
Divisie Endocrinologie
C7-Q
071-5262997
email : i.m.jazet@lumc.nl

PROTOCOL MANAGEMENT

Short title	Effect of Mirabegron on brown adipose tissue
File name	H:\i.m.jazet\mirabegron.doc

INVESTIGATOR SITE

Responsible investigator	Dr. I.M. Jazet
Co-investigator(s)	Dr. M.R. Boon<br>Prof. dr. P.C.N. Rensen<br>Drs. K.J. Nahon<br>Drs. L.G.M. Janssen<br>Dr. J. Burakiewicz<br>Dr. H.E. Kan<br>Prof. A. Webb
Independent Physician	Dr. N. van der Kleij
Study location	Leiden University Medical Centre<br>C2-48, research unit<br>Albinusdreef 2<br>2333 ZA Leiden<br>The Netherlands<br>Tel: 071-5264327

PROJECT TEAM

Clinical	I.M. Jazet<br>M.R. Boon<br>K.J. Nahon
Nursing	E.J.M. Ladan-Eygenraam

LABORATORY	NAME/ADRESS	CONTACT PERSON
Chemistry	CKCL LUMC	B.E.P.B Ballieux
PHARMACY	NAME/ADRESS	CONTACT PERSON
	LUMC. LO-P	L. van der Hulst
Radiology	LUMC	J. Burakiewicz

Protocol Signature Sheet

Name	Signature	
Dr. I.M. Jazet		
Dr. M.R. Boon		
Prof. dr. P.C.N. Rensen		

Drs. K.J. Nahon		
Drs. L.G.M. Janssen		
Dr. J. Burakiewicz		
Dr. H.E. Kan		
Prof. A. Webb		

TABLE OF CONTENTS

LIST OF ABBREVIATIONS 6

SYNOPSIS 7

1. INTRODUCTION AND RATIONALE 11

2. OBJECTIVES 14

3. SUBJECTS 15
   3.1. Population Base 15
      3.1.1. Power calculation 15
   3.2. Inclusion Criteria 15
   3.3. Exclusion criteria 15

4. ETHICAL CONSIDERATIONS 17
   4.1. Regulatory statement 17
   4.2. Recruitment and consent 17
   4.3. Compensation for Injury 17

5. STUDY DESIGN AND RANDOMISATION 18
   5.1. Trial Design 18
   5.2. Randomisation, blinding and treatment allocation 18
   5.3. Withdrawal of individual subjects 18
   5.4. Replacement of individual subjects after withdrawal 18
   5.5. Follow up of subjects withdrawn from treatment 18
   5.6. Incidental finding 18

6. STUDY PROCEDURE 20
   6.1. Schematic overview study 20
   6.2. Screening 20
   6.3. Study Procedure 21
   6.4. Precautions and Restrictions 24
   6.5. Adverse Events 24
   6.6. Post Study Follow-up Visit 25

7. STUDY METHODS 26
   7.1. Indirect calorimetry with ventilated hood 26
   7.2. Blood sampling 26
   7.3. Blood sample handling 27
   7.4. Laboratory tests 27
   7.5. Personalized cooling procedure 27
   7.6. Body temperature distribution 28
   7.7. Finapres 28
   7.8. MRI scan 28

8. STUDY MEDICATION 29
   STUDY 1. 29
   8.1. Name and description of medication/products used in the study 29
   8.2. Potential risks of the medication/products used in the study 29
   8.3. Description and justification of route of administration and dosage 30
   8.4. Summary of dosages and method of administration 30

8.5. Preparation and labelling of treatments 30
8.6. Drug accountability 30
8.7. Procedures for monitoring subject compliance 30

9. ENDPOINTS, DATA ANALYSIS AND PUBLICATION 31
9.1. Endpoints of the study 31
9.2. Data handling and record keeping 31
9.3. Data analysis 31
9.4. Statistical analysis 32

10. ADMINISTRATIVE ASPECTS, MONITORING AND PUBLICATION 33
10.1. Data handling and storage of data, documents and bodily material 33
10.2. Monitoring and quality assurance 33
10.3. Amendments 33
10.4. Annual progress report 34
10.5. End of study report 34
10.6. Data Public disclosure and publication policy 34

11. REFERENCES 35

Appendix A. Overview study day 1 .....

Appendix B. Overview study day 2 & 3 .....

Appendix C. Blood sampling handling .....

LIST OF ABBREVIATIONS

ATP	Adenosine triphosphate
BAT	Brown adipose tissue
cAMP	Cyclic AMP
FDG	Fluorodeoxyglucose
(F)FA	(Free) fatty acids
FT	Facultative thermogenesis
fT4	Free thyroxine
LUMC	Leiden University Medical Centre
METC	Medical Ethical Committee
NST	Non-shivering thermogenesis
PET-CT	Positron emission tomography-computed tomography
PKA	cAMP-dependent protein kinase A
REE	Resting energy expenditure
MRBG	Mirabegron
(S)AE	(Serious) adverse event
SPC	Summary of product characteristics
T2D	Type 2 diabetes mellitus
TC	Total cholesterol
TG	Triglyceride

SYNOPSIS

Rationale

Obesity and type 2 diabetes (T2D) are emerging problems worldwide. In particular South Asian individuals (representing 20% of the world population) have an increased risk of obesity and related disorders. They are at higher risk for the development of T2D as compared to white Caucasians and develop T2D at a younger age and with lower BMI (1,2). The underlying mechanisms that might explain these ethnical differences have not been clarified or understood yet. As a consequence, treatment options are limited and unfocussed, and novel specific strategies are needed.

Brown adipose tissue (BAT) has recently been discovered as a major player in energy metabolism in humans. In a process known as thermogenesis, BAT takes up fatty acids (FA) and glucose from the circulation and subsequently combusts FA and glucose into heat, thereby increasing energy expenditure and improving glucose and FA metabolism (3-5). Using ^{18}F-fluorodeoxyglucose (^{18}F-FDG) (positron emission tomography/computed tomography) PET-CT scan analysis we have recently shown that South Asian individuals have less brown adipose tissue (BAT) than white Caucasians (6). This might suggest that they have a lower energy metabolism, which could underlie their increased predisposition for obesity and the development of T2D.

Activation of BAT, for example by cold exposure, was shown to have beneficial metabolic effects in humans. Cold acclimatization can increase BAT volume, nonshivering thermogenesis, glucose uptake by BAT, as well as decrease fat mass in healthy young men (7-9). Therefore activation of BAT is considered as a novel therapeutic target in the treatment of obesity and T2D(10). As cold exposure is not the most desired therapeutic strategy for humans, current pre-clinical research focuses on pharmacological activation of BAT.

β3-receptor agonists can be used to mimic sympathetic innervation of BAT. Our recent studies using mice with a human-like lipoprotein profile showed that treatment with a β3-receptor agonist decreased fat mass, improved dyslipidemia, increased insulin sensitivity and even attenuated the development of atherosclerosis (11). Likewise, the novel β3-receptor agonist (Mirabegron) has recently been shown to activate BAT in healthy young men as effectively as cold exposure (12). Therefore, ß3-receptor agonism would be a promising treatment option to activate BAT and enhance energy expenditure, especially for South Asians.

Currently the most common way to visualize BAT in humans is by ^{18}F-FDG PET-CT scan. However this method is both expensive and invasive, as it uses ionizing radiation. Recently, MRI, which has no radiation burden, has emerged as a novel method to visualize BAT in humans (13). Activation of BAT results in combustion of intracellular lipid stores, which eventually leads to a lower triglyceride (TG) content. MRI can measure TG content of tissue, and using MRI technology the activation of BAT can be quantified by the relative reduction in the TG content of BAT. The use of MRI to visualize and quantify BAT activity is a safe, cost-effective and innovative alternative to PET-CT, which has a potential to become a new gold standard in the nearby future.

To investigate whether β3-receptor agonism has therapeutic potential to improve the metabolic phenotype of South Asians, we will perform a randomized cross-over study in which 20 healthy young men aged 18-30 years with a lean body type (BMI <25 kg/m^2) are included. Dutch South Asian individuals (n=10) and matched Dutch white Caucasian individuals (n=10) will participate in a cross-over study consisting of three different regimes. In between the different Study days (day 1, 2 and 3) there is a wash-out period of 13 days, which is more than five times the half-life of Mirabegron.

On study day 1 we will measure BAT activity and volume before and after cold exposure. Subject will undergo a baseline MRI scan, to measure BAT volume and activity. In addition, a baseline oxycon and finapres measurement will be done to analyze resting energy expenditure (REE), and blood pressure and heart rate. After this, subjects will be cooled based on an individualized cooling protocol in which maximum non-shivering thermogenesis is reached. After two hours of cold exposure BAT volume and activity will be measured using a second MRI scan and energy expenditure will be analyzed again. If there is no increased BAT activity upon cold stimulation the subject will be excluded from further participation in the study. If there is detectable BAT activity on study day 1, subjects will participate in study days 2 and 3 and will be randomized to receive first Mirabegron or placebo to minimize bias.

On study day 2 the subject will receive a single dose of 200 mg Mirabegron (or placebo). First, REE at baseline will be analyzed using a ventilated hood system and heart rate and blood pressure will be measured using a finapres. Thereafter the compound is administered. At 3.5 hours after the administration of the compound BAT activity and volume will be determined using MRI and energy expenditure will be analyzed again. Furthermore, plasma lipid and catecholamine concentrations will be monitored. Study day 3 is exactly the same as study day 2, except this time the subject will receive the other treatment (either 200 mg Mirabegron or Placebo).

This study will investigate whether β3-receptor agonism has therapeutic potential to improve the metabolic phenotype of South Asians. The effects of a β3-receptor agonist on BAT activity in South Asians have never been studied before. Elucidating the effects of this β3-receptor agonist on BAT activity in South Asians might have major clinical implications, as it might result in the discovery of a potential novel treatment strategy to combat obesity and T2D in this especially vulnerable population.

Objectives:
Primary objective: To evaluate the effect of Mirabegron treatment on BAT activity measured by MRI in South Asians compared with white Caucasians.

Secondary objectives:

1. To assess differences in the effect of Mirabegron treatment on a) REE, b) plasma lipid levels c) and sympathetic output between South Asian and white Caucasian individuals.
2. To assess the differences between indirect BAT activation by cold exposure and direct BAT activation with Mirabegron treatment
3. To assess the MRI scan as novel way to visualize BAT activity
4. To assess the effect of mild cold exposure and Mirabegron on plasma lipoprotein profiles

Study Design:	Randomized cross-over study
Planned Sample:	20 male, lean body type, 18-30 year old. Dutch South Asian individuals (n=10) and matched Dutch white Caucasian individuals (n=10)
Drugs and Dosages:	Mild cold exposure, one single dose of Mirabegron (200 mg) and placebo
Main Parameters:	

- BAT volume and activity: MRI scan
- Energy expenditure: indirect calorimetry (correlation with BAT activity measured via MRI scan)
- Anthropometric: Weight, height and fat percentage as measured by BIA
- Lipid metabolism: Total cholesterol (TC), TG, free fatty acids (FFA) and lipoprotein panel in plasma
- Sympathetic outflow: Plasma catecholamine concentrations
- Supraclavicular temperature: iButtons
- Finapres Blood pressure, heart rate, pulse rate variability

<u>Study procedure:</u>
In the current study, the effect of one single dose of Mirabegron (200 mg) versus placebo and cold-exposure will be studied in health young (18-30 years) Dutch South Asian (n=10) and Dutch Caucasian (n=10) men.

All study subjects will be screened. If the subject meets all the inclusion criteria, is willing to participate in the study and has signed the informed consent, he will be included. All subjects will be asked not to make any changes in their usual diets and physical activities before the start of the whole study.

At screening a thorough medical history (see F1.1) and physical examination will be performed. Subjects will be examined while in the fasting state. Anthropometric measurements will be performed as well as a BIA measurement for determination of body fat percentage and basal blood sample will be taken by means of a venapunction. Basal blood measurements include kidney, liver, thyroid, hemoglobulin, natrium, kalium, ureum and lipid parameters as well as glucose concentrations.

If subjects are eligible to participate they first will complete Study day 1, on which we will measure BAT activity and volume before and after cold exposure (see Appendix A for an overview of the study day). Subject will undergo a baseline oxycon and finapres measurement to determine REE and blood pressure and heart rate (30 min). After this a baseline MRI scan will be made followed by an individualized cooling protocol so that the maximum non-shivering thermogenesis is reached. During the cold exposure, skin temperature will be measured via ‘iButtons’. One iButtons will be placed under the armpit as an approximation of the ‘core temperature’. When shivering temperature is reached, stable cooling period will start (t=0). After 30 minutes of stable cold exposure (t=30) REE and blood pressure and heart rate will be analyzed a second time using a ventilated hood system and the finapres. Thereafter (t=60) BAT activity and volume will be measured using a second MRI scan. Furthermore, during the cooling procedure a venous blood sample will be obtained every 15 minutes to monitor dynamic changes in plasma lipids. In addition, plasma catecholamine concentrations will be determined. If there is no increased BAT activity upon cold stimulation the subject will be excluded from further participation in the study. If there is detectable BAT activity on Study day 1, subjects will participate in Study days 2 and 3 and will be randomized to receive first Mirabegron or placebo to minimize bias.

On study day 2 first baseline oxycon and finapres measurements will be performed to determine REE and blood pressure and heart rate (30 min). Thereafter, the subject will receive a single dose of 200 mg Mirabegron (four tables of 50 mg) (or placebo) (t=0). Again skin temperature will be measured via ‘iButtons’. One iButton will be placed under

the armpit as an approximation of the ‘core temperature’. After 1, 2 and 3 hours (t=60, t=120 and t=180) REE is analyzed using a ventilated hood system and blood pressure and heart rate are monitored using the finapres. Thereafter, 3.5 hours after the administration of the compound (t=210) BAT activity and volume will be determined using an MRI scan. Furthermore, a venous blood sample will be drawn every 15 minutes to monitor changes in plasma lipids. In addition, plasma catecholamine concentrations will be determined.Study day 3 is exactly the same as study day 2, except this time the subject will receive the other compound (either 200 mg Mirabegron or placebo) (see Appendix B for an overview of study days 2 and 3).

All study days will take place at the Leiden University Medical Centre (LUMC). Between study days 2 and 3 a wash-out period of 13 days will be maintained to make sure that the drug is out of the body during the next exam.

Study outline

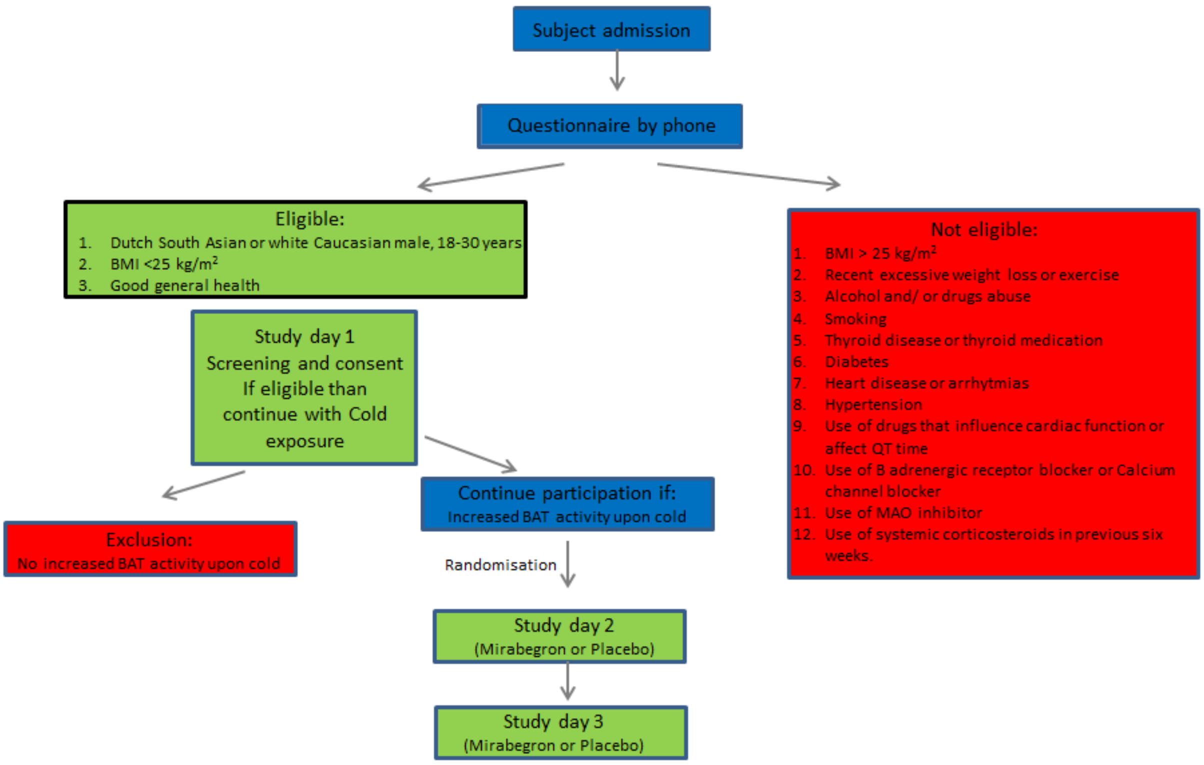

1. INTRODUCTION AND RATIONALE

1.1 Introduction

Obesity is a major public health problem, affecting many people worldwide. Obesity is known to be one of the main factors in the development of type 2 diabetes and other complications, such as cardiovascular disease. Since the incidence of obesity and type 2 diabetes is increasing at an alarming rate and effective treatments are lacking, novel treatment strategies are needed.

Ethnicity was shown to be an important risk factor for the development of obesity. Especially in South Asian individuals a disadvantageous metabolic profile - including abdominal obesity, dyslipidemia and insulin resistance - is highly prevalent (1,2). As a consequence, they are at higher risk for the development of T2D as compared to white Caucasians, and develop T2D at a younger age and with lower BMI. The underlying mechanism of this increased predisposition for the development of T2D in South Asians is poorly understood, but their lower energy metabolism is likely to play an important part.

BAT has recently been discovered as a major factor in energy metabolism in humans. In a process known as thermogenesis, BAT takes up FA and glucose from the circulation and subsequently combusts FA and glucose into heat, thereby increasing energy expenditure and improving glucose and fat metabolism(3-5). Using ^{18}F-FDG PET-CT scan analysis, we have recently shown that South Asian individuals have less BAT than white Caucasians (6). In addition, it has been shown that obese individuals have lower BAT activity as compared to lean individuals. Therefore the activation of BAT is considered as a potential novel therapeutic target in the treatment of obesity and T2D.

BAT is strongly innervated by the sympathetic nervous system and the most potent stimulator is cold exposure, resulting in release of noradrenalin (NA) from sympathetic nerve endings, which binds to β3-adrenergic receptors on BAT thereby enhancing thermogenesis(3). Cold acclimation increases BAT volume, non-shivering thermogenesis, glucose uptake by BAT, and decreases fat mass in healthy young men. Prolonged cold exposure has too many disadvantages to be considered a viable treatment option. However, ß3-receptor agonists can be used to mimic sympathetic innervation of BAT. Our recent studies using mice with a human-like lipoprotein profile showed that treatment with a ß3-receptor agonist decreased fat mass, improved dyslipidemia, increased insulin sensitivity and attenuated the development of atherosclerosis(11). Likewise, β3-receptor agonism has recently been shown to activate BAT in healthy young men as effectively as cold exposure(12). Therefore, ß3-receptor agonism would be a promising treatment option to activate BAT and enhance energy expenditure, especially for South Asians, as there have less BAT and are therefore prone to develop obesity and T2D.

1.2 BAT: function, localization

Mammalian adipose tissue consists of different subtypes of which the two most well-known are white adipose tissue (WAT) and brown adipose tissue (BAT). WAT is widely known as an organ that can store fatty acids in the form of triglycerides (TG) in the case of a positive energy balance. In contrast, BAT burns fatty acids for the production of heat, thereby contributing as a major factor in energy metabolism. BAT is strongly innervated by the sympathetic nervous system and the most potent stimulator is cold exposure. Upon cold stimulation, the hypothalamus induces sympathetic neurons to release NA. NA stimulates β3-adrenergic receptors on BAT, thereby activating an intracellular pathway, which eventually leads to activation of mitochondrial uncoupling protein 1 (UCP-1). UCP-1 is a protein located in the inner membrane of the mitochondria and facilitates uncoupling of

electron transport from adenosine triphosphate (ATP) synthesis. Consequently, oxygen consumption is no longer coupled to ATP synthesis and instead heat is generated. This process is known as non-shivering thermogenesis (NST). NST is especially important for maintenance of core body temperature in neonates who are very prone to develop hypothermia due to their large body surface area. Until recently, it was thought that BAT disappeared with increasing age and was not present anymore in adult humans. However, this vision was refuted when FDG for PET-CT scans showed that upon exposure to cold symmetrical areas of glucose uptake appeared that corresponded to BAT, thus indicating that BAT is still present and metabolically active in the adult human body. Human BAT is located along the large vessels and in the supraclavicular area. In this way, the heat produced by BAT can be spread throughout the body. Humans have an estimated 100-200 g of active BAT, which accounts for approximately 15% of total energy expenditure. Therefore, modulation of BAT volume and activity is considered a promising target to treat and prevent the development of obesity and obesity-related disorders, such as T2D.

1.3 BAT visualization

Currently the most common way to visualize BAT in humans is by ^{18}F-FDG PET-CT scan. After injection of a radioactive labeled glucose tracer (^{18}F-FDG), metabolically active tissues can be visualized with a PET-CT scan as FDG is taken up by the sodium-independent glucose transport family (likely GLUT1, GLUT3 and GLUT4) and is primarily taken up by metabolically active tissues, including BAT. The tracer is picked up along with the glucose. However this method is both expensive and invasive, as it uses ionizing radiation. Recently, MRI, which has no radiation burden, has emerged as a novel method to visualize BAT in humans (13). Activation of BAT results in combustion of intracellular lipid stores, which eventually leads to a lower (TG content. MRI can measure TG content of tissue, and using MRI technology the activation of BAT can be quantified by the relative reduction in the TG content of BAT (Figure 1). The use of MRI to visualize and quantify BAT activity is a safe, cost-effective and innovative alternative to PET-CT, which has a potential to become a new gold standard in the nearby future.

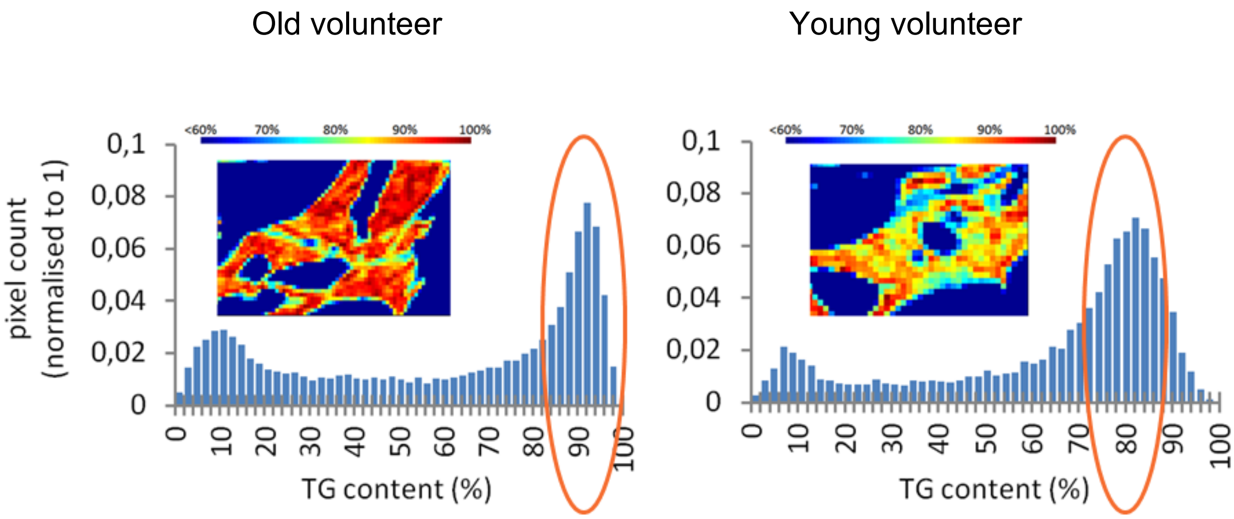

Figure 1. TG content measurements in two healthy volunteers of different age. Older volunteer (50 years old) has a higher TG content, indicating less BAT, while the younger volunteer (25 years old) shows lower average TG content, indicating more BAT.

1.4 Pharmacological activation of BAT

BAT is strongly innervated by the sympathetic nervous system and the most potent stimulator is cold exposure, resulting in release of noradrenalin (NA) from sympathetic nerve endings, which binds to β3-adrenergic receptors on BAT, thereby enhancing thermogenesis. Prolonged cold exposure, however, is not the most suitable treatment option. ß3-receptor agonists can be used to mimic sympathetic innervation of BAT. Our recent studies using mice with a human-like lipoprotein profile showed that treatment with a ß3-receptor agonist decreased fat mass, improved dyslipidemia, increased insulin sensitivity and even attenuated the development of atherosclerosis(11). Likewise, β3-receptor agonism has recently been shown to activate BAT in healthy young men as effectively as cold exposure (12). Therefore, ß3-receptor agonism would be a promising treatment option to activate BAT and enhance energy expenditure, especially for South Asians, who have less BAT and are therefore prone to develop obesity and T2D.

Mirabegron has recently been identified as a novel oral selective ß3-receptor agonist. Mirabegron shows high intrinsic activity for ß3-adrenoreceptor and very low intrinsic activity for ß1- and ß2-adrenoceptors and is tested in phase 3 trials for the treatment of overactive bladder. After stimulation of the ß3-receptor it couples via Gs proteins to adenylyl cyclase, which results in an increase of intracellular cyclic AMP (cAMP) levels and a subsequent activation of cAMP-dependent protein kinase A (PKA). In addition, it is already known that activation of cAMP and PKA in brown adipocytes is involved in the stimulation of thermogenesis, as it eventually leads to activation of UCP1. Therefore BAT activation by Mirabegron is likely mediated via enhanced PKA and as a consequence upregulated UCP1 in the inner membrane of the mitochondria which in turn stimulates uncoupling in mitochondria thereby increasing thermogenesis.

In conclusion, BAT likely contributes to NST. Mirabegron is a novel ß3-receptor agonist which has already shown to activate BAT in healthy young men as effectively as cold exposure. South Asians have less active BAT, which might underlie their increased predisposition for the development of obesity and related diseases, such as T2D. Therefore South Asians, who possess a disadvantaged metabolic profile, might benefit from this compound even more than Caucasians. With this study will not only give more insight into the effects of the β3-receptor agonist on BAT activity in South Asians, it might also lead to a potential novel treatment strategy to combat obesity and T2D in this especially vulnerable population. The use of validation of MRI to visualize BAT and quantify BAT activity is a safe, cost-effective and innovative alternative to PET-CT, which has a potential to become a new gold standard in the nearby future.

2. OBJECTIVES

Primary objective: To evaluate the effect of Mirabegron treatment on BAT activity measured by MRI in South Asians compared with white Caucasians.

Secondary objectives:

1. To assess differences in the effect of Mirabegron treatment on a) REE, b) plasma lipid levels c) and sympathetic output between South Asian and white Caucasian individuals.
2. To assess the differences between indirect BAT activation by cold exposure and direct BAT activation with Mirabegron treatment
3. To assess the MRI scan as novel way to visualize BAT activity
4. To assess the effect of mild cold exposure and Mirabegron on plasma lipoprotein profiles

3. SUBJECTS

3.1. Population Base

The study will be carried out in 20 health young men, 18-30 year old. 10 Dutch South Asian and 10 Dutch white Caucasian individuals.

3.1.1. Power calculation

For this study, the main outcome parameters is BAT activity. Up till now, only one study is available in literature that assessed the effect of a cold intervention on this parameter using the MRI (14) The authors showed that cold exposure (12°C during 90 minutes) resulted in 4% decreased in triglyceride (TG) content of BAT (SD= 1.7%) in Caucasians, indicating an increased activity of BAT. We expect the same effect for Mirabegron in white Caucasians as it directly targets the β3-adrenergic receptors. Therefore, for white Caucasians, we consider a decrease in TG content of BAT by Mirabegron of 4% as therapeutically relevant as this is as effective as cold exposure.
For South Asians it is previously shown with PET-CT scan (6) that they have less BAT activity upon mild cooling compared to white Caucasians. Therefore, in South Asians, we expect a decrease in TG content of BAT by cold of 1.8%. However, for Mirabegron we expect to reach the same BAT activity in South Asians as in white Caucasians because of the direct stimulation of the β3-adrenergic receptors. Therefore in South Asians, we expect a decrease in TG content of BAT by Mirabegron of 4%.

Thus, with an SD of 1.7, $\alpha = 0.05$, $\beta = 80\%$ we need 10 subjects per group (https://www.sealedenvelope.com/power/continuous-superiority/).

3.2. Inclusion Criteria

- Male volunteers. 10 white Caucasians, born in the Netherlands. 10 South Asians, living in the Netherlands.
- Age: 18-30 years
- BMI ≤ 25 kg/m^2

3.3. Exclusion criteria

- BMI > 25 kg/m^2
- Recent excessive weight loss or exercise
- Alcohol and/ or drugs abuse
- Smoking
- Any significant chronic disease, including diabetes
- Renal, hepatic or endocrine disease
- Heart disease or arrhythmias
- Thyroid disease or thyroid medication
- Hypertension
- Use of medication known to influence glucose and/or lipid metabolism or BAT activity (*e.g.* beta blockers or calcium channel blockers)
- Use of drugs that influence cardiac function or affect QT time
- Use of MAO inhibitor
- Use of systemic corticosteroids in previous six weeks

- Recent participation in other research projects (within the last 3 months), participation in 2 or more projects in one year
- Contraindications for undergoing an MRI scan:
    - Presence of non-MR safe metal implants or objects in the body.
    - Pacemaker, neurostimulator, hydrocephalus pump, drug pump, non-removable hearing aid, large recent tattoos.
    - Claustrophobia
    - Tinnitus or hyperacusis

To make sure participants do not meet any of the exclusion criteria we will ask them to complete a questionnaire (Medical checklist, see F1.1, MR screening form, see F1.2) and draw blood (16.0 mL) for analysis of plasma glucose, hemoglobulin, natrium, kalium, ureum and lipid levels, and parameters of liver, kidney and thyroid function during the screening session. In addition, body weight and height will be measured to calculate BMI and a BIA measurement will be performed to determine body fat percentage.

4. ETHICAL CONSIDERATIONS

4.1. Regulatory statement

The study will be conducted according to the principles of the "Declaration of Helsinki" (as amended in Fortaleza, Brazil, October 2013) and in accordance with the Guideline for Good Clinical Practice (CPMP/ICH/135/95 - July 2002).

4.2. Recruitment and consent

The protocol of this study will be submitted to the Medical Ethics Committee (METC) of the LUMC. The study will not commence before formal approval has been granted. Subjects will be given oral and written explanation (see section E2 about the study. After they have given written acknowledgement of informed consent to participate, a medical screening will take place. After approval by the subjects (see section E2), their general practitioners will be notified. Although the subjects will be told they are free to leave the study at any time, it will be attempted to recruit subjects that are likely to continue the study to completion.

In addition to careful medical supervision, volunteers will be paid €150.- for the whole study. A proportional payment will be made when the volunteer leaves on his own wish, and a full payment for the study will be made when the subject is asked by the investigators to discontinue the study for medical reasons. In addition, travel expenses will be repaid, €0.19/km will be repaid if subjects travel by car, public transport will be restituted on basis of second class.
Subjects will be recruited via advertisements (see section E3) placed in local media and public places.

4.3. Compensation for Injury

Patients will be insured by a no-fault insurance of the LUMC (see section G1).

5. STUDY DESIGN AND RANDOMISATION

5.1. Trial Design

Cross-over study, with three different treatment regimes.

5.2. Randomisation, blinding and treatment allocation

After the medical screening and mutual agreement of participation in the study, patients will be included in the study. Patients will first all undergo study day 1, in which they will be exposed to cold. If they have detectable BAT upon cold exposure they can also participate in study day 2 and 3 in which we will compare they effects on BAT of 200 mg of a β3-receptor agonist (Mirabegron) administrated as four tablets of 50 mg (packed in capsules to mimic the placebo capsules) compared with placebo in random order. Beforehand an unblinded pharmacist will set up a list in which each study subject number is coupled to a box number. The pharmacy then gives the medication according to the randomisation list. Therefore, the study will be conducted double blind. Furthermore, the staff performing the MRI analyses and the laboratory measures only get samples (or the scans) with a subject and occasion number on it.

5.3. Withdrawal of individual subjects

Subjects can leave the study at any time for any reason if they wish to do so without any consequences. The responsible investigator can also withdraw a subject if continuing participation is in his opinion deleterious for the subject's well-being. Subjects can also be withdrawn in case of protocol violations and non-compliance. When a subject withdraws from the study a medical examination will be performed. In case of withdrawal because of a severe or serious adverse event (SAE), haematological, blood chemistry and urine laboratory tests or other special examinations may be performed.

5.4. Replacement of individual subjects after withdrawal

Dropouts will be replaced by newly recruited subjects.

5.5. Follow up of subjects withdrawn from treatment

After a subject's withdrawal, he will be followed at the research laboratory C2-47 by the responsible investigator as frequently as necessary. If necessary, another specialist will be consulted or the patient will be referred to a specialist for further treatment or investigation.

5.6. Incidental finding

Since the study will involve an MRI of the chest there is a possibility of incidental findings occurring. All relevant images will be reviewed by a trained

radiologist, and any potential irregularities will be referred to the subject's general practitioner.

6. STUDY PROCEDURE

6.1. Schematic overview study

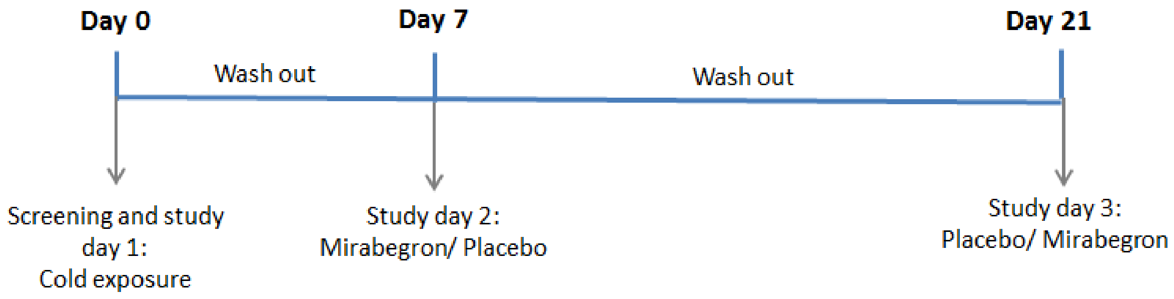

6.2. Screening

Eligibility will be assessed with a telephone interview. During this telephonic interview the study protocol will be briefly explained to the subjects and some of the basic inclusion criteria will be checked. These include weight, height, BMI, gender, age, fitness level, medication use and if the subject has thyroid disease or T2DM, as well as ability to undergo an MRI scan. When this is in compliance with our study protocol, subjects will receive the detailed subject information (see section E1) via e-mail or mail. They will be instructed to read it carefully and to ask questions when things are unclear. After a week of reflection, the subjects are invited to the LUMC to our research unit where the researcher will again explain the study to them and answer their questions. If they want to participate after completely understanding all study procedures, they can sign the informed consent form and will participate in the screening.

The screening procedure will include:

- Medical history questionnaire (see F1.1) and MRI screening form (see F1.2) that will be filled in by subjects together with the investigator.
- Body weight as measured using a digital balance (E1200, August Sauter GmBH, Albstadt, Germany), and body height.
- BIA measurement as measure of body fat percentage.
- Blood sample: a fasting blood sample will be taken via venapunction to determine fasting blood glucose and lipid levels, hemoglobulin, natrium, kalium, ureum and parameters of liver (ASAT, ALAT, γ-GT), kidney (creatinine), and thyroid (fT4) function.

When the subject meets all the inclusion criteria, is willing to participate in the study, and has signed the informed consent, he will be included in the study.

6.3. Study Procedure

When subject meets the inclusion criteria they can immediately continue with the first occasion where we assess the BAT response after cold exposure. If the BAT response is sufficient the subjects can participate in study days 2 and 3 to determine the effect of Mirabegron or placebo on BAT volume and activity, REE and plasma lipid and catecholamine levels. At these three study days, all measurements will be performed in the morning under fasted conditions. This means that subjects are not allowed to drink (except water) and eat from 10 pm the evening before, until arrival in the morning. Furthermore, subjects will be asked:

- not to exercise 48 hours in advance
- not to drink alcohol 24 hours in advance
- not to drink tea or coffee 24 hours in advance
- to eat a standardized meal the evening before (options will be given by the researcher)

Description of treatment

The first part of the study is an unblinded measurement, as it would be difficult to cool the subject in a blinded fashion. First subject will undergo a baseline oxycon and finapres measurement to determine REE and blood pressure and heart rate (30 min). Thereafter a MRI scan is made to measure the TG content of BAT under thermoneutral circumstances. The MRI scan takes between 30-40 minutes. Next the subject will be exposed to a personalized cooling protocol. The subject will be cooled down gradually by lying between two mattresses with cold water flowing through them. If the subject start shivering the temperature will be adjusted a few degrees higher to maximize non-shivering thermogenesis and the stable cold periode starts (t=0). During the cold exposure, skin temperature will be measured via 'iButtons'. One iButtons will be placed under the armpit as an approximation of the 'core temperature'. After 30 minutes of stable cold exposure (t=30) REE will be analyzed again using a ventilated hood system and blood pressure and heart rate will be monitored using a finapres. Thereafter (t=60) a MRI scan will be performed to analyze BAT volume and activity. If the subjects show increased BAT activity upon cold, they are eligible to participate in the second part of the study. The second part has a randomized placebo-controlled double blind design, i.e. each subject will be randomized to receive either a single dose of Mirabegron (200 mg p.o. administrated as four tables of 50 mg packed in capsules) or placebo first. The compounds will be packed and labeled by the pharmacy and provided in non-subject specific numbered boxes so that the subject nor the investigator will know which compound is administered (double blind). Labeling will be in accordance with Annex 13, local law and trial requirements. An example of the label of the compound is provided in D3. The pharmacy will do the randomization of the study and give out the boxes according to the randomization schedule when a new patient enters the trial. During the study day a physician will administer the drug to the subject an watch the ingestion of the drug to ensure compliance.

Study day 1 (= screening and cold exposure)

Shortly after arrival (8:00 AM), a medical screening will be performed. The

medical screening includes measurement of length, body weight, body fat percentage, blood pressure, a venous blood sample and a medical questionnaire. If the subject is eligible to participate, a oxycon and finapres measurement are performed to asses REE and blood pressure and heart rate at baseline. Thereafter a MRI scan will be made to determine TG content in BAT under thermoneutral circumstances. Next, an intravenous cannula will be inserted to collect blood samples during the study days. In addition, wireless iButtons will be placed on the skin to monitor skin temperature. One iButtons will be placed under the armpit as an approximation of the ‘core temperature’. Thereafter the individualized cooling protocol will start. The subject will lay in a bed between two water-perfused matrasses and water temperature will be gradually decreased to the point that shivering occurs (from our previous study we know that this occurs at a water temperature of approximately 14°C)(6). After the onset of shivering, temperatures are stabilized just above shivering level to induce maximal NST. From this point on (t=0), a one-hour cooling period will start. During the cooling period, blood pressure, heart rate, and shivering are monitored at fixed intervals. In addition, a venous blood sample (22,5 mL) will be drawn before the start of the personalized cooling and after 1 hour of stable cooling. Also regular blood samples (3,5 mL) will be drawn from the cannula to monitor lipoprotein levels during cold exposure. At t=30, after 30 minutes of stable cooling an oxycon measurement will be performed to determine energy expenditure and a finapres measurement will be performed to determine blood pressure and heart rate. At t=60 min, after one hour of stable cooling, the MRI scan is performed to visualize BAT. The scanning protocol takes between 30-40 minutes. After the MRI scan, a lunch will be provided and the subject can go home.

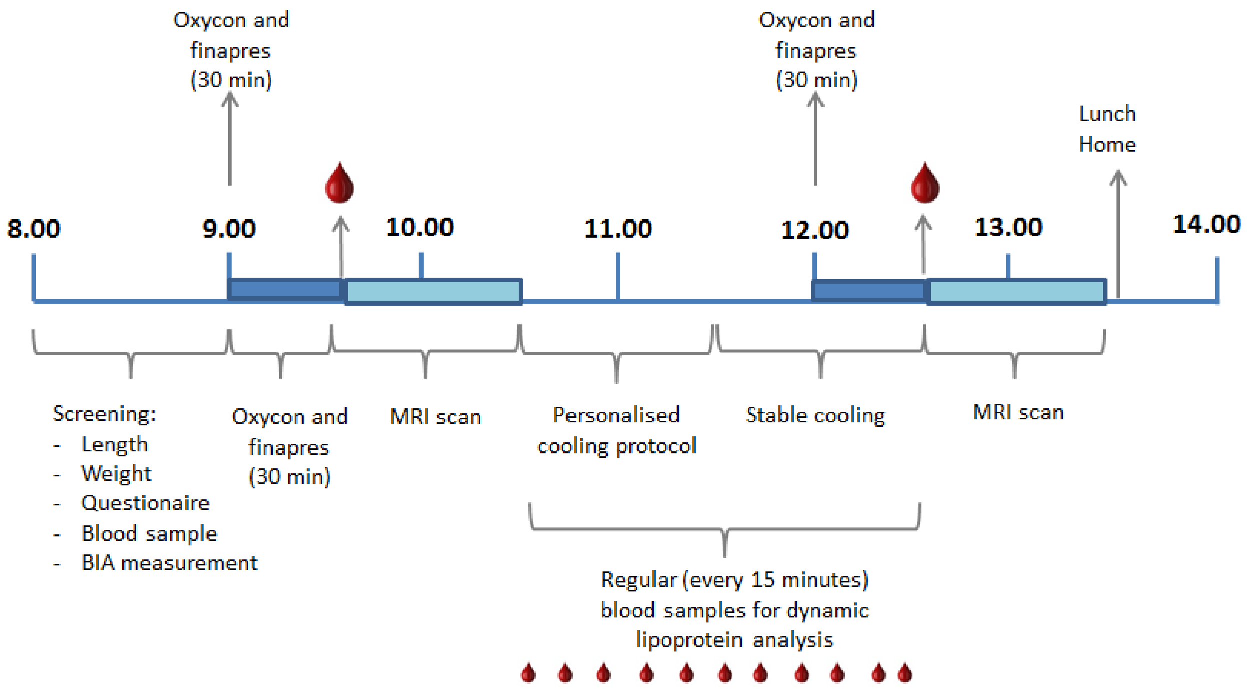

Day 2 & 3 (= Mirabegron or placebo)

Upon arrival (approx 8:00 AM), an intravenous cannula will be inserted and wireless iButtons will be placed on the skin to monitor skin temperature. One iButtons will be placed under the armpit as an approximation of the 'core temperature'. Next, baseline REE and blood pressure and heart rate will be determined using the oxycon and finapres. Thereafter the drug (ie a single dose of 200 mg Mirabegron administered as four tablets of 50 mg packed in capsules or placebo) will be administered orally (t=0). A venous blood sample (22,5 mL) will be drawn before the administration of the drug and 3,5 hours after ingestion (at t=210). In addition, between t=0 and t=210 several venous blood samples (3,5 mL) will be taken to analyse dynamics of plasma lipoproteins. Also, REE and blood pressure and heart rate will be monitored using an oxycon andfinapres at regular intervals after administration of the drug (t=60, t=120 and t=180). Thereafter, at t=210, three and a half hours after ingestion of the drug the MRI is made to visualize and quantify BAT. The scanning protocol takes between 30 and 40 minutes. After the MRI scan, a lunch will be provided and the subject can go home. In between occasion 2 and 3 there is a wash-out period of 13 days minimally, to ensure that the drug is eliminated from the body.

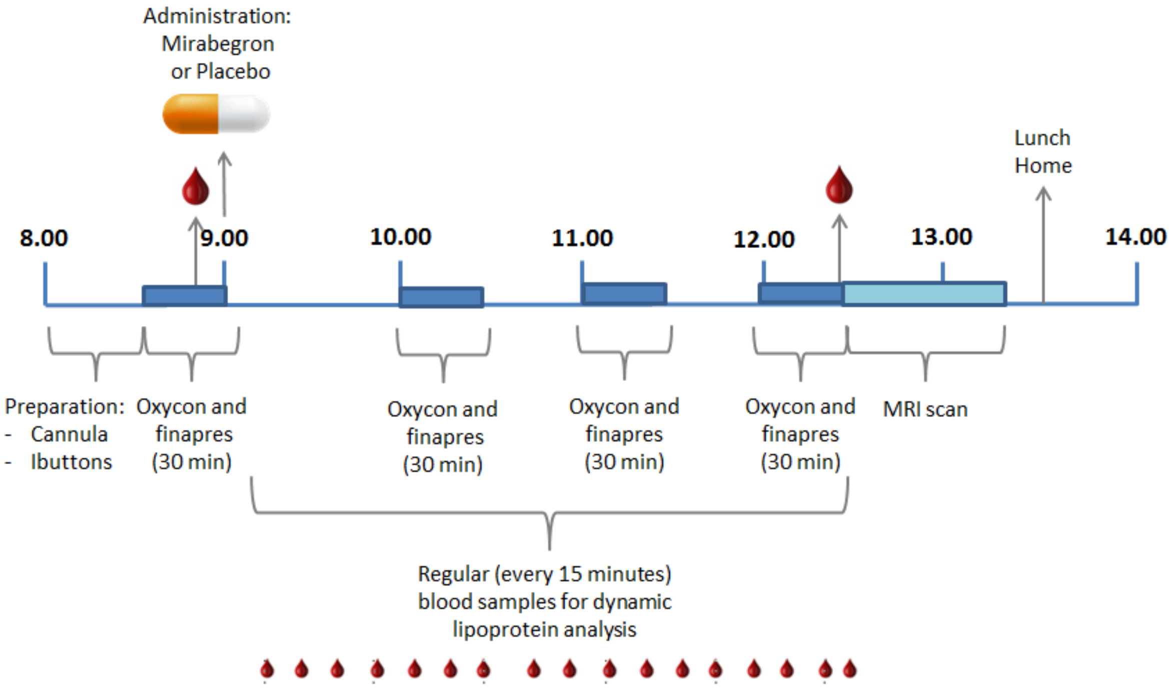

6.4. Precautions and Restrictions

The most frequently reported side-effects of Mirabegron are tachycardia, headache and urinary tract infections. Tachycardia and headache were reported at a similar incidence compared with placebo treatment. During this study the subjects will receive only 1 dosage of Mirabegron 200 mg administered as four tables of 50 mg packed in capsules to mimic the placebo capsules.

During the whole study day a physician and an assistant will be present at the subject´s bedside.

To prevent undercooling, the subject will be monitored and asked every 15 minutes whether he is experiencing shivering. If so, temperature will be adjusted accordingly.

6.5. Adverse Events

Adverse events (AEs) are defined as any undesirable experience occurring to a subject during a clinical trial, whether or not considered related to the used medication or the infused drugs. All AEs reported spontaneously by the subject or observed by the investigator or his/her staff will be recorded on the AE data collection form. The intensity of these AEs will be graded by the investigator as follows:

Mild:	Discomfort noted but no disruption of normal daily activity
Moderate:	Discomfort sufficient to reduce or affect normal daily activity
Severe:	Inability to work or perform daily activity

The chronicity of the event will be classified by the investigator on a three-item scale as defined below:

Single occasion:	Single event with limited duration
Intermittent:	Several episodes of an event, each of limited duration
Persistent:	Event that remains indefinitely

For each AE, the relationship to the used medication or infused drug (definite, probable, possible, unknown, definitively not) as judged by the investigator, will be recorded, as well as any actions undertaken on behalf of the AE, will be recorded. The occurrence of an AE that is fatal, life-threatening, disabling or requires in-patient hospitalization, or causes congenital anomaly, will be described according to CPMP guidelines as “serious” AEs and will be notified in writing to the METC.
All AEs will be followed until they have abated, or until a stable situation has been reached. Depending on the event, follow up may require additional tests or medical procedures as indicated.

6.6. Post Study Follow-up Visit

A follow-up phone call will be planned 1 week after the study. General well-being and possible side effects will be evaluated.

7. STUDY METHODS

In this section all measurements, as briefly described in the section above, will be described in detail.

7.1. Indirect calorimetry with ventilated hood

Patients will be placed under the ventilated hood three times in total. The effects on REE will be monitored via indirect calorimetry using the ventilated hood system. Subjects will lie on a bed in a quiet room, for 30 minutes and must stay awake during the procedure. Measurements of total CO_2 production (= VCO_2) and O_2 consumption (= VO_2) will be made in order to measure substrate oxidation rates (glucose and lipid oxidation) (15) These measurements are not expected to give any side effects.

7.2. Blood sampling

At screening, a total amount of 16,0 mL blood will be taken for determination of glucose, creatinine, ASAT, ALAT, γ-GT, hemoglobulin, serum free T4 (fT4), total cholesterol (TC) and triglyceride (TG), natrium, kalium, ureum. Most chemistry can be determined in a SST®II Advance Gel tube of 8.5 mL, all from Becton and Dickinson®. Hemoglobulin will be determined in EDTA vacutainer (3,5 mL) from Becton and Dickinson®. Natrium, kalium and ureum will be determined in Heparin vacutainer (4,0 mL) from Becton and Dickinson®.

Before cooling and after 2 hours of stable cooling blood samples will be collected to determine TC, FFA, TG, HDL, LDL, glucose, insulin, cortisol, ACTH, norepinephrine /epinephrine and RNA. In addition, during the individualized cooling procedure on study day every 15 minutes a venous blood sample will be collected from the cannula for lipoprotein analysis. In total of 9 samples will be collected during a period of 2 hours and collected into a SST®II Advance Gel tube of 3,5 mL from Becton and Dickinson®. (total amount of blood drawn during this study day: 76 mL). We will determine a set of lipoproteins in the these frequent samples.

During study day 2 and 3 blood samples will be collected before administration of the compound and before the MRI scan to determine TC, FFA, TG, HDL, LDL, glucose, insulin, cortisol, ACTH, norepinephrine/epinephrine and RNA. In addition, every 15 minutes during 3.5 hours, starting from the administration of the drug a venous blood sample will be collected from the cannula for lipoprotein analysis. In total 15 samples will be collected into a SST®II Advance Gel tube of 3,5 mL from Becton and Dickinson® (total amount of blood drawn: 97,5 mL). We will determine a set of lipoproteins in the these frequent samples.

In total, during this study 287.5 mL of blood will be withdrawn.

7.3. Blood sample handling

After the blood has been collected, all tubes (except the serum tubes) will be immediately put on ice. All blood samples, except serum samples, will be centrifuged promptly (2,000 g at 4°C, during 10 minutes) and subsequently plasma and serum will be divided in separate plastic tubes as mentioned below and frozen (-80°C) until assay. The serum samples have to coagulate before centrifugation, this will be established after 20-30 minutes after which centrifugation and subsequent storage in plastic tubes will take place. For the noradrenalin measurement, before centrifugation 330 µL of glutathione (0.5%) will be added. The PAXgene tube for RNA analysis will be stored at room temperature. For a complete overview of the blood sample handling, see Appendix C.

7.4. Laboratory tests

Fasting serum glucose, TC, TG, creatinine, ASAT, ALAT will be measured at the laboratory for Clinical Chemistry at the LUMC, a Modular P800 analyzer (Roche, Mannheim, Germany). fT4 will be measured using an ECLIA on a Modular Analytics E170 analyzer (Roche Diagnostics, Mannheim, Germany). Serum FFA will be determined spectrophotometrically using validated assay kits (Boehringer, Mannheim) in the Endocrinology laboratory at the LUMC. Serum noradrenalin will be measured in collaboration with Erasmus MC. The NMR lipoprotein panel will be measured in collaboration with the Genome Analysis Center of the Helmholtz Zentrum München, Germany.

7.5. Personalized cooling procedure

Since cold exposure is currently the golden standard to activate BAT, a cooling procedure is applied before we analyse the TG content of BAT using the MRI. Subjects will be cooled using a dedicated cooling device (Blanketrol® III, Cincinnati Sub-Zero (CSZ) Products, Inc), which is provided to us kindly by FMH Medical B.V. (the Netherlands) free of charge for the duration of 1 year. These cooling devices are used frequently on intensive care units, and also in our previous study (16) as well as by our collaborator prof. dr. W.D. van Marken Lichtenbelt (Maastricht University). During the cooling procedure subjects will stay in a clinical examination room kept at basal conditions (21°C); they will be placed in a supine position on a bed to lay comfortable. To exclude the artefact of muscle activity, subjects will be instructed to lay still. To keep conditions as uniform as possible, all subjects will wear boxer shorts only. During the cold exposure, skin temperature will be measured via 'iButtons'. One iButtons will be placed under the armpit as an approximation of the 'core temperature'.
During the cooling procedure subjects will be exposed to mild cold (approx. 16°C, a temperature at which the subject will start shivering) for 2 hours. Since the onset temperature of shivering shows a high interindividual variation, we will use a personal cooling protocol to ensure maximum non-shivering thermogenesis (and thus an equal maximum activation of BAT). The right temperature will be determined via a subjective method, *e.g.* to ask the subject if he experiences shivering. The time needed to achieve the right temperature is approximately 60 minutes (6). Then the official cooling period

is started (t=0 min). After one hour of stable cold exposure (i.e. a condition of maximal NST; t=60 min) the MRI scan is made to visualize TG content of BAT.

7.6. Body temperature distribution

Skin temperatures will be measured by wireless iButton dataloggers (iButton®) (see Product information D2.1). Mean skin temperature is calculated using skin temperatures measured at the 14 ISO-defined skin sites (17). One iButtons will be placed under the armpit as an approximation of the ‘core temperature’.

7.7. Finapres

Blood pressure, heart rate and pulse variability will be monitored using a finapres (see Product information D2.2).

7.8. MRI scan

Subjects will undergo four MRI scans in total, 2 on the first day and one each on days 2 and 3. All the scans will be performed on a 3T Philips Ingenia MRI system at the LUMC in collaboration with Dr. J. Burakiewicz, Dr. H.E. Kan and Prof. A. Webb. The scanning involves the subject lying still in supine position for a period of approximately 30 to 40 minutes, with an MR coil placed on the torso. Care will be taken to place the subject in the same position in all the scans; this will be done by asking the volunteer to lower his shoulders as much as possible for each scan, with hand palms against the body. We will use a 3D 6-point chemical-shift-encoded gradient-recalled echo (6-point Dixon) (18). In addition a T2-weighted reference scan will be done for localization purposes. The sequence has been extensively tested in healthy volunteers and shown to reliably identify BAT deposits (see Fig. 1). Other previously tested sequences used in clinics or research sites around the world may also be added to the protocol to extract further more detailed information about BAT deposits; however the total scan time will not exceed 1 hour.

8. STUDY MEDICATION

STUDY 1.

8.1. Name and description of medication/products used in the study

- Mirabegron: β3-receptor agonist will be ordered from Astellas BV. The tablets will be put in a capsule by the pharmacy of the LUMC to mimic the placebo capsules.For more information about the compound please see the summary of product characteristics (SPC) information on Mirabegron (see D2).
Mirabegron-placebo will be facilitated by the pharmacy of the LUMC.

8.2. Potential risks of the medication/products used in the study

Mirabegron: See also the D2 with product information.
The most frequently reported side-effects of Mirabegron are tachycardia, nausea and urinary tract infections. The most common drug-related side effect is mild tachycardia and headache, although these were reported at a similar incidence compared with placebo treatment. For safety our research physician is available for questions 24 hours daily.

Cooling procedure: During study day 1 a cooling procedure is used to activate BAT, in which the subject is exposed to mild cold (14°C) for 2 hours. During this time the subject will be asked every 15 minutes whether he is experiencing shivering. If so, temperature will be adjusted accordingly. Cooling is done using a special cooling device, also used on intensive care units. No side effects are expected.

Indirect calorimetry: The ventilated hood, used for indirect calorimetry, is a transparent light-weighted plastic hood, which will be placed over the subject's head for twenty-thirty minutes during all three study days. Unless the patient is claustrophobic no side effects are expected to occur.

Anthropometry: The anthropometric measurements are simple, non-invasive procedures, not expected to give side effects.

Finapres: The is a non-invasive method to measure blood pressure and heart rate, not expected to give side effects.

BIA measurement: the BIA measurement will be used to body fat percentage. No side effects are expected.

MRI: the MRI scan will be used to determine TG content of BAT. Unless the patient is claustrophobic no side effects are expected to occur.

8.3. Description and justification of route of administration and dosage

Mirabegron: Mirabegron will be delivered as compressed film-coated tablets of 50 mg. Subjects have to take four tablets of 50 mg orally. These tablets will be repacked into capsules to mimic the placebo capsules.

Miabegron-placebo, four capsules of placebo orally.

8.4. Summary of dosages and method of administration

Mirabegron: Four tables of 50 mg per os, packed as capsules

Mirabegron-placebo: Four capsules per os.

8.5. Preparation and labelling of treatments

Mirabegron and is manufactured, packed and labelled by Astellas BV. The tablets are repacked into capsules by the pharmacy of the LUMC to resemble the placebo.The placebo is facilitated by the pharmacy of the LUMC. Randomisation will be done by the pharmacy as well. Labeling will be in accordance with Annex 13, local law and trial requirements. The examples of labels are not readily available, but will be supplied when ready.

8.6. Drug accountability

Mirabegron: The QP release and drug accountability will be cared for by the Department of Clinical Pharmacy of the LUMC. The trial product will be dispensed to each subject as required according to treatment group by the clinical pharmacist. The subject only gets a single dose during the study day. The ingestion of the drug will be conducted under supervision of a physician to ensure compliance. No trial product will be dispensed to any person not enrolled in the trial.

8.7. Procedures for monitoring subject compliance

Mirabegron: The tablets will be given to the subject under the supervision of the physician or research assistant. In addition it is only one single dose per treatment, therefore compliance is expected to be maximal.

9. ENDPOINTS, DATA ANALYSIS AND PUBLICATION

9.1. Endpoints of the study

We aim to detect differences in all of the following study endpoints between Mirabegron and placebo treatment:

Primary endpoint:
- BAT activity measured by MRI scan

Secondary endpoints
- Fasted REE by indirect calorimetry
- Change in supraclavicular skin temperature (as measured with iButtons)
- Fasting serum markers for lipid metabolism (TC, HDL-C, LDL-C, TG, free fatty acids) and dynamic changes in lipoproteins.
- Fasting serum markers for sympathetic output (norepinephrine, epinephrine)

9.2. Data handling and record keeping

Data will be recorded on data collection forms and will be entered after validation in a computer system for subsequent tabulation and statistical analysis. The data will be handled confidentially.

9.3. Data analysis

Indirect calorimetry

The oxycon provides values of respiratory quotient (RQ), O2 consumption (VO_2) and CO_2 production (VCO_2) every 60 seconds. Measurements will be performed during 30 minutes. The average values of each measurement will be calculated after removing the values obtained during the first 5 minutes. Subsequently, total lipid and carbohydrate oxidation can be calculated with the following equations:

Glucose oxidation = $4.12\ VCO_2 – 2.91\ VO_2 – 2.33\ N$

Lipid oxidation = $1.69\ VO_2 – 1.69\ VCO_2 – 2.03\ N$

REE is calculated from:

REE = $3.82\ VO_2 + 1.23\ VCO_2 –1.94\ N$

MRI scan:

Data analysis will be performed using custom developed software. BAT repository will be manually segmented by a trained operator and TG content calculated in the volume according to the formula:

$$TG = \frac{F}{F + W} \times 100\%$$

where F and W are measured fat and water quantities. This calculation will be

done on a voxel-by-voxel basis and the overall TG content will be estimated by averaging the values in the volume of interest.

Temperature registration:
iButtons: The output value given by the iButton is the instantaneous skin temperature at that particular moment. During the measurements the information is stored in a protected memory section (NV RAM). It can afterwards be transferred to a personal computer for data analysis. For this purpose, the iButton will be clipped into an adapter connected to the computer (19).

9.4. Statistical analysis

Data will be presented as mean ± standard error of the mean. Comparisons between response after administration of cold versus Mirabegron versus placebo, will be made by using the paired Student's *t*-test. Nonparametric (Wilcoxon signed-rank test) tests for paired samples will be performed when appropriate. Comparisons between response after in South Asians and white Caucasians, will be made by using the unpaired Student's *t*-test. A p-value of < 0.05 will be considered statistically significant.

10. ADMINISTRATIVE ASPECTS, MONITORING AND PUBLICATION

10.1. Data handling and storage of data, documents and bodily material

Data will be collected in case report forms. Original datafiles will be stored in locked closets. Data will be digitally encrypted before they are stored, including personal information. Personal information regarding participants will be anonymized upon inclusion and participants will be attributed a study number. The study number will be used to mark data as well as bodily material. The personal data will remain confidential. Only the principal researchers will have access to decryption documents. The data and documents will be stored in the LUMC or the LUMC external archive for the duration of 15 years after the termination of the study. The bodily material will be stored in the LUMC for the duration of 15 years for future use. Participants will be asked for permission to use bodily material for additional research in the future. Upon withdrawal of this consent, the material will be destroyed.

10.2. Monitoring and quality assurance

The risk classification according to the NFU risk assessment is negligible. Therefore the monitoring can be performed by a BROK certificated colleague who is not involved in this research project. He/she will monitor the study once a year. He will check the inclusion rate, informed consent forms of the first 10% of the subjects, the trial master file, the instruction to the subjects, the study standard operating procedures S)AEs and suspected unexpected serious adverse reactions.

10.3. Amendments

Amendments are changes made to the research after a favourable opinion by the accredited METC has been given. All amendments will be notified to the METC that gave a favourable opinion.

10.4. Annual progress report

The sponsor/investigator will submit a summary of the progress of the trial to the accredited METC once a year. Information will be provided on the date of inclusion of the first subject, numbers of subjects included and numbers of subjects that have completed the trial, serious adverse events/serious adverse reactions, other problems and amendments.

10.5. End of study report

The sponsor will notify the accredited METC and the competent authority of the end of the study within a period of 90 days. The end of the study is defined as the last patient's last visit.

In case the study is ended prematurely, the sponsor will notify the accredited METC and the competent authority within 15 days, including the reasons for the premature termination.

Within one year after the end of the study, the investigator/sponsor will submit a final study report with the results of the study, including any publications/abstracts of the study, to the accredited METC and the Competent Authority.

10.6. Data Public disclosure and publication policy

This study will be registered in a public trial registry. There are no disclosures.

11. REFERENCES

Reference List

1. Misra A, Shrivastava U: Obesity and dyslipidemia in South Asians. *Nutrients* 5:2708-2733, 2013

2. Symonds ME: Ethnicity and its effects on brown adipose tissue. *Lancet Diabetes Endocrinol* 2:185-186, 2014

3. Boon MR, Bakker LE, Meinders AE, van Marken LW, Rensen PC, Jazet IM: [Brown adipose tissue: the body's own weapon against obesity?]. *Ned Tijdschr Geneeskd* 157:A5502, 2013

4. Geerling JJ, Boon MR, van der Zon GC, van den Berg SA, van den Hoek AM, Lombes M, Princen HM, Havekes LM, Rensen PC, Guigas B: Metformin lowers plasma triglycerides by promoting VLDL-triglyceride clearance by brown adipose tissue in mice. *Diabetes* 63:880-891, 2014

5. Stanford KI, Middelbeek RJ, Townsend KL, An D, Nygaard EB, Hitchcox KM, Markan KR, Nakano K, Hirshman MF, Tseng YH, Goodyear LJ: Brown adipose tissue regulates glucose homeostasis and insulin sensitivity. *J Clin Invest* 123:215-223, 2013

6. Bakker LE, Boon MR, van der Linden RA, Arias-Bouda LP, van Klinken JB, Smit F, Verberne HJ, Jukema JW, Tamsma JT, Havekes LM, van Marken Lichtenbelt WD, Jazet IM, Rensen PC: Brown adipose tissue volume in healthy lean south Asian adults compared with white Caucasians: a prospective, case-controlled observational study. *Lancet Diabetes Endocrinol* 2:210-217, 2014

7. van der Lans AA, Hoeks J, Brans B, Vijgen GH, Visser MG, Vosselman MJ, Hansen J, Jorgensen JA, Wu J, Mottaghy FM, Schrauwen P, van Marken Lichtenbelt WD: Cold acclimation recruits human brown fat and increases nonshivering thermogenesis. *J Clin Invest* 123:3395-3403, 2013

8. Nedergaard J, Cannon B: The browning of white adipose tissue: some burning issues. *Cell Metab* 20:396-407, 2014

9. Saito M, Okamatsu-Ogura Y, Matsushita M, Watanabe K, Yoneshiro T, Nio-Kobayashi J, Iwanaga T, Miyagawa M, Kameya T, Nakada K, Kawai Y, Tsujisaki M: High incidence of metabolically active brown adipose tissue in healthy adult humans: effects of cold exposure and adiposity. *Diabetes* 58:1526-1531, 2009

10. Hanssen MJ, Hoeks J, Brans B, van der Lans AA, Schaart G, van den Driessche JJ, Jorgensen JA, Boekschoten MV, Hesselink MK, Havekes B, Kersten S, Mottaghy FM, van Marken Lichtenbelt WD, Schrauwen P: Short-term cold acclimation improves insulin sensitivity in patients with

type 2 diabetes mellitus. *Nat Med* 21:863-865, 2015

11. Berbee JF, Boon MR, Khedoe PP, Bartelt A, Schlein C, Worthmann A, Kooijman S, Hoeke G, Mol IM, John C, Jung C, Vazirpanah N, Brouwers LP, Gordts PL, Esko JD, Hiemstra PS, Havekes LM, Scheja L, Heeren J, Rensen PC: Brown fat activation reduces hypercholesterolaemia and protects from atherosclerosis development. *Nat Commun* 6:6356, 2015

12. Cypess AM, Weiner LS, Roberts-Toler C, Franquet EE, Kessler SH, Kahn PA, English J, Chatman K, Trauger SA, Doria A, Kolodny GM: Activation of human brown adipose tissue by a beta3-adrenergic receptor agonist. *Cell Metab* 21:33-38, 2015

13. van Rooijen BD, van der Lans AA, Brans B, Wildberger JE, Mottaghy FM, Schrauwen P, Backes WH, van Marken Lichtenbelt WD: Imaging cold-activated brown adipose tissue using dynamic T2*-weighted magnetic resonance imaging and 2-deoxy-2-[18F]fluoro-D-glucose positron emission tomography. *Invest Radiol* 48:708-714, 2013

14. Vanessa Stahl1, Florian Maier1 Ralf O. Floca2 Moritz C. Berger1 Mauricio Berriel Diaz3 Martin T. Freitag2 Marc-André Weber4 Antonia Dimitrakopoulou-Strauss5 and Armin M. Nagel. In vivo Assessment of Cold Stimulation Effects on the Fat Fraction of Brown Adipose Tissue using Dixon MRI. Proc. Intl. Soc. Mag. Reson. Med. 23. 2015.

15. Simonson DC, DeFronzo RA: Indirect calorimetry: methodological and interpretative problems. *Am J Physiol* 258:E399-E412, 1990

16. Bakker LE, Boon MR, van der Linden RA, Arias-Bouda LP, van Klinken JB, Smit F, Verberne HJ, Jukema JW, Tamsma JT, Havekes LM, van Marken Lichtenbelt WD, Jazet IM, Rensen PC: Brown adipose tissue volume in healthy lean south Asian adults compared with white Caucasians: a prospective, case-controlled observational study. *Lancet Diabetes Endocrinol* 2:210-217, 2014

17. van Marken Lichtenbelt WD, Daanen HA, Wouters L, Fronczek R, Raymann RJ, Severens NM, Van Someren EJ: Evaluation of wireless determination of skin temperature using iButtons. *Physiol Behav* 88:489-497, 2006

18. Hu HH, Wu TW, Yin L, Kim MS, Chia JM, Perkins TG, Gilsanz V: MRI detection of brown adipose tissue with low fat content in newborns with hypothermia. *Magn Reson Imaging* 32:107-117, 2014

19. van Marken Lichtenbelt WD, Daanen HA, Wouters L, Fronczek R, Raymann RJ, Severens NM, Van Someren EJ: Evaluation of wireless determination of skin temperature using iButtons. *Physiol Behav* 88:489-497, 2006
